# Adenylate Cyclase 5 (Adcy5) Deficiency Impairs Pigment Granule Dispersion in Melanophores and Erythrophores in Nile Tilapia

**DOI:** 10.3390/cells15151347

**Published:** 2026-07-27

**Authors:** Jia Sun, Peng Li, Jiawen Yao, Yu He, Hao Liu, Siyu Ju, Hongsheng Shi, Xingyong Liu, Deshou Wang

**Affiliations:** Key Laboratory of Freshwater Fish Reproduction and Development (Ministry of Education), Key Laboratory of Aquatic Science of Chongqing, Integrative Science Center of Germplasm Creation in Western China (Chongqing) Science City, School of Life Sciences, Southwest University, Chongqing 400715, China; sunjiaaa@email.swu.edu.cn (J.S.); pl199110043@126.com (P.L.); g093615@email.swu.edu.cn (J.Y.); hy17785221307@email.swu.edu.cn (Y.H.); swu_ih@foxmail.com (H.L.); 17362494986@163.com (S.J.); 15912022469@163.com (H.S.)

**Keywords:** Adcy5, pigment dispersion, melanophore, erythrophore, cAMP-PKA signaling pathway, melanin synthesis

## Abstract

**Highlights:**

**What are the main findings?**

- Comparative genomic analyses revealed conserved synteny and protein domains of Adcy5.
- Loss of *adcy5* eliminated black vertical bars and impaired pigment granule dispersion in melanophores and erythrophores.

**What are the implications of the main findings?**

- cAMP treatment partially rescued melanosome dispersion in *adcy5* mutant melanophores.
- Adcy5 is a conserved regulator of chromatophore pigmentation in Nile tilapia.

**Abstract:**

Adenylate cyclase 5 (Adcy5) generates cyclic adenosine monophosphate (cAMP) downstream of G protein-coupled receptor signaling, yet its role in vertebrate pigmentation remains incompletely understood. Here, we generated a CRISPR/Cas9-mediated *adcy5* knockout line in Nile tilapia (*Oreochromis niloticus*) to investigate its function in chromatophore biology. Loss of *adcy5* resulted in a pronounced disruption of body coloration, characterized by the absence of vertical black bars and a global reduction in pigmentation. Despite this, chromatophore number was largely unaffected, indicating that Adcy5 is not required for pigment cell specification but is essential for functional pigmentation. At the cellular level, pigment granules in melanophores and erythrophores failed to undergo dispersion and instead remained constitutively aggregated, revealing a primary defect in intracellular pigment granule transport. Consistently, *adcy5* mutants exhibited reduced expression of key melanogenesis-associated genes, including *mitfa*, *tyrb*, *tyrp1a*, and *tyrp1b*, accompanied by decreased melanin content across multiple tissues. Pharmacological activation of cAMP signaling partially rescued the pigment dispersion defect, whereas stimulation of upstream α-MSH signaling produced only limited effects, placing Adcy5 upstream of intracellular cAMP production within the pigment regulatory cascade. Importantly, we further demonstrate that Adcy5 is required for erythrophore pigment granule dispersion, extending its functional role beyond melanophore biology. Together, these findings identify Adcy5 as a conserved regulator integrating cAMP-dependent pigment synthesis and granule transport across multiple chromatophore types in teleost fish.

## 1. Introduction

The adenylate cyclase (ADCY) family proteins are key membrane-associated enzymes regulated by G protein-coupled receptors (GPCRs) [1,2,3]. By catalyzing the conversion of ATP into the second messenger cyclic adenosine monophosphate (cAMP) [2], ADCYs regulate diverse physiological processes, including signal transduction, gene expression, biosynthesis, and intracellular transport [3,4]. In vertebrates, body coloration is a complex trait shaped by ecological adaptation and sexual selection, relying on the coordinated development and function of multiple chromatophore types, pigment synthesis, and intracellular transport of pigment granules [5,6]. As a central regulator of intracellular cAMP production, ADCY-mediated signaling is therefore considered essential for understanding the cellular mechanisms underlying vertebrate pigmentation.

Among ADCY isoforms, Adcy5 has emerged as an important regulator of pigmentation in both mammalian and teleost systems. In vitro experiments demonstrate that loss of Adcy5 impairs proliferation of mouse B16F10 melanoma cells [7,8]. However, Adcy5 knockout mice exhibit no obvious pigmentation defects, suggesting substantial functional redundancy among ADCY family members in mammals. In contrast, studies in teleosts have demonstrated a more prominent role for Adcy5 in pigment patterning. In guppy, *adcy5* mutation abolishes melanosome dispersion and disrupts stripe formation [9]. Similarly, zebrafish *adcy5* mutants exhibit partial defects in melanosome dispersion, whereas *adcy3a* single mutants display little or no visible phenotype [10,11,12]. By contrast, simultaneous disruption of *adcy3a* and *adcy5* causes severe melanosome aggregation accompanied by complete loss of stripe patterning, demonstrating partial functional redundancy while identifying Adcy5 as the predominant regulator of melanophore pigment dispersion. Together, these findings establish Adcy5 as a core component of cAMP-dependent pigment granule transport in teleost melanophores.

Despite these advances, previous studies have focused almost exclusively on melanophores, and whether Adcy5 regulates other chromatophore types remains unknown. cAMP–PKA signaling has also been implicated in pigment granule transport in xanthophores and erythrophores in several teleost species [13,14,15]. Moreover, Adcy5-related signaling has been linked to regulation of *mitf*, a master transcription factor controlling chromatophore development [16,17]. In Nile tilapia, disruption of the *mitf* pathway affects both xanthophores and erythrophores [18], suggesting that upstream regulators such as Adcy5 may exert broader functions across multiple chromatophore types. However, this hypothesis has not been experimentally tested. Importantly, although classical teleost models such as zebrafish have greatly advanced our understanding of melanophore biology, they lack true erythrophores, limiting investigation of red pigment granule transport. By contrast, Nile tilapia (*Oreochromis niloticus*), which possesses melanophores, xanthophores, erythrophores, and iridophores, provides an ideal system for investigating whether Adcy5 coordinates pigment granule dynamics across multiple chromatophore types [19].

In this study, we generated an *adcy5* knockout line in Nile tilapia and investigated its role in chromatophore function through phenotypic characterization, gene expression profiling, and pigment quantification. We aimed to determine whether Adcy5 is required not only for melanophore pigmentation but also for erythrophore pigment granule dispersion. By extending the functional analysis of Adcy5 beyond melanophores, this study provides new insights into the cellular mechanisms underlying chromatophore regulation and offers a molecular framework for understanding pigmentation traits in teleosts, with potential implications for pigmentation trait improvement in aquaculture species.

## 2. Materials and Methods

### 2.1. Animals

The Nile tilapia (*Oreochromis niloticus*) used in this study were provided by the Key Laboratory of Freshwater Fish Resources and Development (Ministry of Education) at Southwest University (Chongqing, China). All fish were maintained at 26 °C in recirculating aquaculture systems under natural photoperiod conditions. Embryos were generated by artificial fertilization, and fertilized eggs were incubated at 26 °C in a recirculating water incubator to obtain fry. All animal experiments were conducted in strict compliance with the Guide for the Care and Use of Laboratory Animals and were approved by the Experimental Animal Ethics Committee of Southwest University.

### 2.2. Phylogenetic Analyses

A maximum-likelihood (ML) phylogenetic tree was constructed using the amino acid sequences of Adcy5 from selected species, including Nile tilapia, Japanese medaka, torafugu, zebrafish, spotted gar, chicken, common lizard, human, coelacanth, elephant shark, lamprey, lancelet, oyster, trichoplax, swiftwater hydra, fruit fly, and vase tunicate. The analysis was performed using PhyloSuite v2 [20]. The phylogenetic tree was visualized and edited using iTOL [21], and further modified using Adobe Illustrator 2020 for figure preparation. Bootstrap values indicate the support for each branch based on 1000 replicates.

The GenBank accession numbers of the Adcy5 amino acid sequences of all species used in this study are listed in Table S1.

### 2.3. Synteny Analysis

The chromosomal locations and orientations of vertebrate *adcy5* genes and their neighboring genes were obtained from the NCBI database and visualized using Adobe Illustrator 2020.

### 2.4. Adcy5 Amino Acid Sequence Alignment Analysis

The full-length amino acid sequences of Adcy5 from Nile tilapia, Japanese medaka, zebrafish, spotted gar, coelacanth, tropical clawed frog, common lizard, chicken, and mouse were extracted using TBtools-II software (version 2.224). Multiple sequence alignment was performed using DNAMAN 7.0.2. Residues showing ≥50% identity at each aligned position were highlighted. Functional domains were predicted using the SMART database (http://smart.embl-heidelberg.de/, accessed on 3 July 2026).

### 2.5. Quantitative Real-Time PCR (qPCR)

#### 2.5.1. General qPCR Procedure

Total RNA was extracted from Nile tilapia samples using RNAiso reagent (Takara, Kasukabe, Japan). RNA concentration was measured using a NanoDrop 2000 spectrophotometer (Thermo Fisher Scientific, Wilmington, DE, USA). First-strand cDNA was synthesized using the PrimeScript^®^ RT reagent kit with gDNA Eraser (Takara, Japan). The synthesized cDNA was diluted 10-fold and used as the template for qPCR. qPCR was conducted according to the manufacturer’s instructions for the TB Green^®^ Premix Ex Taq™ II kit (Takara, Beijing, China). The relative expression levels of target genes were normalized to *β-actin* and calculated using the 2^−ΔΔCt^ method. Unless otherwise stated, three biological replicates and three technical replicates were used for each group. Statistical analysis was performed using GraphPad Prism 9 software. Primer sequences used for qPCR are listed in Table S2.

#### 2.5.2. Expression Pattern Analysis of Adcy5 in Nile Tilapia

To determine the expression profile of *adcy5*, total RNA was extracted from adult tissues, including brain, pituitary, eye, gill, heart, liver, spleen, intestine, kidney, ovary, testis, muscle, head kidney, and skin. Total RNA was also extracted from whole larvae at 3–10 days post-fertilization (dpf). qPCR was performed as described above.

#### 2.5.3. qPCR Analysis of Genes Involved in Melanophore Differentiation and Melanogenesis

Melanocyte stem cells (MSCs) residing in the dorsal root ganglia are established during embryogenesis. Upon activation by specific signals, they migrate and begin to express *mitfa*, thereby giving rise to melanophore precursors. In zebrafish pigment mutants such as *picasso*/*erbb3b*^−/−^ and *sparse*/*kita*^−/−^, defects in MSC establishment lead to a significant reduction in adult melanophore numbers. Given the roles of Erbb3b and Kit signaling in melanophore stem cell fate determination, and the convergence of Kita- and Kitb-mediated pathways on Mitfa during Nile tilapia pigmentation [22], we examined *erbb3b*, *kita*, *mitfa*, and melanogenesis-related genes in *adcy5*^−/−^ mutants by qPCR. Total RNA was extracted from wild-type (WT) and *adcy5*^−/−^ mutants at 8 and 120 dpf. qPCR was performed as described above. cDNA from 8 dpf samples was used to quantify the expression of *erbb3b*, whereas cDNA from 120 dpf skin samples was used to analyze the expression of *kita*, *mitfa*, *tyrb*, *tyrp1a*, and *tyrp1b*.

### 2.6. Generation of adcy5 Homozygous Mutants

A target site (GGGACCGAACGTAGCGTACGTGG) on the first exon of the *adcy5* gene was designed using the online tool CRISPRdirect (http://crispr.dbcls.jp/, accessed on 3 July 2026), with TGG serving as the protospacer adjacent motif (PAM). Cas9 mRNA (1000 ng/µL) and guide RNA (500 ng/µL) were mixed at a 1:1 ratio, and phenol red was added as an indicator at one-tenth of the total volume. The mixture was then injected into zygotes by microinjection to obtain F0 chimeric tilapia *adcy5* mutants. F0 chimeras were outcrossed to obtain F1 progeny. Subsequently, F1 males and females were intercrossed to generate F2 homozygous mutants. The primers used for establishing the line are listed in Table S3.

### 2.7. Body Color Phenotype Analysis

To characterize phenotypic alterations in *adcy5*^−/−^ mutants, WT and homozygous mutant fish of comparable size were selected at 7, 16, 21, 30, 90, and 180 dpf. Fish at or before 30 dpf were observed and imaged using a Nikon SMZ25/SMZ18 stereomicroscope (Nikon, Tokyo, Japan), while those at 90 and 180 dpf were photographed with a Sony ILCE-7SM3 mirrorless camera (Nikon, Tokyo, Japan). Chromatophore morphology in local tissues, including scales, skin, and caudal fin, was captured using the stereomicroscope. For pharmacological analysis, melanin coverage area and the number of melanophores were quantified using Image J Pro 1.51.

### 2.8. Quantification of Melanin

To measure melanin content, skin, scale, and caudal fin tissues (approximately 100 mg each) were collected from adult WT and *adcy5*^−/−^ mutant fish. Each sample was placed into a 1.5-mL microcentrifuge tube containing 1 mL of radioimmunoprecipitation assay (RIPA) buffer and subjected to mechanical disruption. After homogenization, the samples were incubated on ice for 5 min and then centrifuged at 12,000 rpm for 10 min at 4 °C. The supernatant was discarded, and the pellet was resuspended in 1 mL of 1 M NaOH and fully digested at 100 °C. Absorbance at 490 nm was measured using 1 M NaOH as a blank control.

### 2.9. Melanosome Dispersion Assay

To investigate melanosome dispersion, dorsal views of 10-dpf WT and *adcy5*^−/−^ larvae were imaged using a Nikon SMZ25/SMZ18 stereomicroscope. The larvae were then incubated with either 5 mM cAMP for 40 min or 0.25 mM α-MSH for 3 h to ensure maximal pigment dispersion. After treatment, dorsal images were captured again under the same conditions. Pigmented area of individual dorsal melanophores before and after treatment was quantified using Image J software. The longer incubation time for α-MSH treatment was used because of its lower concentration and to determine whether this upstream factor could still induce melanosome dispersion in the absence of functional Adcy5.

### 2.10. Statistical Analysis

Statistical analysis was performed using GraphPad Prism 9 software. Data are presented as the mean ± standard deviation (SD). Differences between two groups were assessed using an unpaired two-tailed Student’s *t*-test. For comparisons among multiple groups, one-way analysis of variance (ANOVA) was used, followed by Tukey’s multiple comparison test. A threshold of *p* < 0.05 was applied to determine statistical significance.

## 3. Results

### 3.1. Synteny Analysis of Adcy5

To investigate the conservation of *adcy5* genomic locus, we performed synteny analyses of the chromosomal regions harboring *adcy5* in Nile tilapia and other representative vertebrates. The results revealed that *adcy5* and its neighboring genes, including *hacd2*, *sec22a*, *pdia5*, *sema5b*, and *slc49a4*, exhibited a highly conserved gene order across the examined vertebrate species. This conserved syntenic arrangement indicates strong evolutionary conservation of the genomic region and suggests that it has experienced relatively few chromosomal rearrangements during vertebrate evolution. Notably, *slc49a4* was not retained within the corresponding *adcy5* syntenic block in chicken and coelacanth, although genome database searches indicate that this gene is present elsewhere in these genomes. This suggests lineage-specific disruption of local synteny rather than complete gene loss. Furthermore, *ccdc14* and *mylk* were highly conserved among the analyzed tetrapods and the closely related coelacanth, whereas *arl5a*, *cacnb4a*, and *stam2* showed high conservation among ray-finned fishes. However, *arl5a* was not retained within this locus in zebrafish, indicating disruption of local synteny in this lineage (Figure S1).

### 3.2. Amino Acid Sequence Alignment and Phylogenetic Analysis of Adcy5

To assess the conservation of Adcy5 among vertebrates, multiple amino acid sequence alignments were performed. The results showed that all examined vertebrate Adcy5 proteins possess two catalytic domains (highlighted by red boxes). The first catalytic domain displayed a high degree of sequence conservation across the selected species, with only a few amino acid substitutions detected. In contrast, the second catalytic domain was comparatively less conserved, particularly in its COOH-terminal region, where substantial sequence divergence was observed. Moreover, pronounced sequence variability was detected in the regions upstream of the first catalytic domain and in the linker region between the two catalytic domains (Figure S2).

To elucidate the evolutionary history of Adcy5, a phylogenetic tree was reconstructed. The analysis revealed that Adcy5 originated as early as *Trichoplax adhaerens* and has remained highly conserved throughout evolution (Figure S3).

### 3.3. The Expression Pattern of Adcy5 in Nile Tilapia

**Figure 1:**
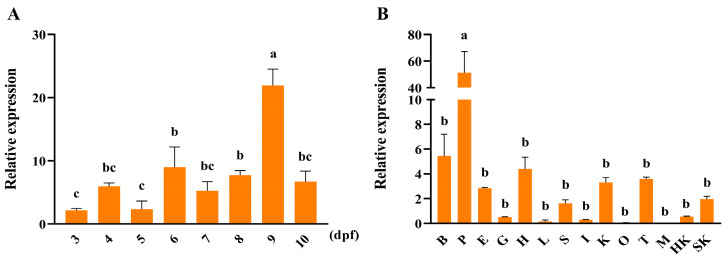
Expression of *adcy5* in different developmental stages of larvae and various tissues of adult fish in Nile tilapia by qPCR. (**A**) Expression of *adcy5* at different developmental stages (3–10 dpf). (**B**) Expression of *adcy5* in various tissues of adult fish. Data are expressed as the mean ± SD (*n* = 3). Significant differences in the data were tested by one-way ANOVA followed by Tukey’s test. Groups with no lowercase letters in common are significantly different (*p* < 0.05), whereas groups sharing at least one letter are not significantly different. B, brain; P, pituitary; E, eye; G, gill; H, heart; L, liver; S, spleen; I, intestine; K, kidney; O, ovary; T, testis; M, muscle; HK, head kidney; SK, skin. dpf, days post-fertilization.

qPCR was performed to analyze the expression patterns of *adcy5* in WT Nile tilapia during larval development (whole embryos at 3–10 dpf) and across various adult tissues. The results showed that *adcy5* expression peaked at 9 dpf during larval development. In adult fish, the pituitary gland exhibited the highest expression level, which was significantly higher than that in all other tissues examined. Relatively high expression was also observed in the brain, heart, testis, and kidney (Figure 1). In contrast, minimal expression was detected in the liver, intestine, ovary, and muscle.

### 3.4. Establishment of adcy5 Homozygous Mutant Lines

**Figure 2:**
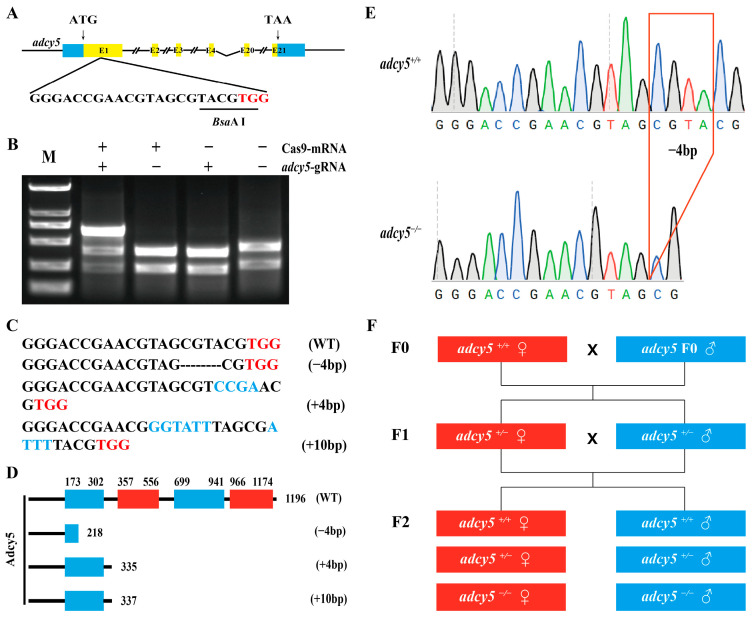
Establishment of *adcy5* homozygous mutants in Nile tilapia. (**A**) Gene structures of *adcy5* in tilapia showing the target sites, PAM sequences, and restriction site of *Bsa*AI, respectively. (**B**) Mutation verification by digestion with restriction endonuclease. (**C**) Mutation types of F1 heterozygotes. The PAM sequence is shown in red, added nucleotides are indicated in blue, and deleted nucleotides are represented by short dashes. (**D**) Amino acid sequence prediction after frameshift mutation. The blue region represents the transmembrane domain, while the red region represents the catalytic domain. (**E**) Verification of *adcy5* homozygous mutants by Sanger sequencing. The dotted lines are the background ruler lines provided by the sequencing software and have no biological significance. (**F**) The establishment processes of *adcy5* homozygous mutants.

To functionally characterize *adcy5* in Nile tilapia, a knockout line was generated using the CRISPR/Cas9 system. A target site containing a *Bsa*AI recognition sequence was designed in the first exon of *adcy5* (Figure 2A). Genomic DNA from F0 embryos was amplified by PCR and subjected to restriction enzyme digestion. Embryos co-injected with *adcy5*-gRNA and Cas9 mRNA exhibited both uncut and cleaved bands, whereas embryos injected with either component alone or uninjected WT controls exhibited only the cleaved band (Figure 2B). F0 founders were crossed with WT individuals to produce the F1 generation, which was subsequently screened by Sanger sequencing. Various indel mutations were identified (Figure 2C), all predicted to cause premature termination of the Adcy5 protein, thereby disrupting the catalytic domains and resulting in a loss-of-function phenotype (Figure 2D). To establish a stable homozygous mutant line, F1 heterozygotes harboring an identical −4 bp deletion were intercrossed to generate F2 homozygous mutants. The genotype of F2 individuals was confirmed by Sanger sequencing (Figure 2E). A schematic summary of the mutant generation strategy is illustrated in Figure 2F.

### 3.5. Phenotypic Analysis of Adcy5 Homozygous Mutants

To characterize the role of *adcy5* in pigmentation, we tracked the phenotypic progression of *adcy5*^−/−^ mutants from larval to adult stages.

#### 3.5.1. Early Developmental Phase (7–30 Dpf): Establishment of Pigment Pattern

**Figure 3:**
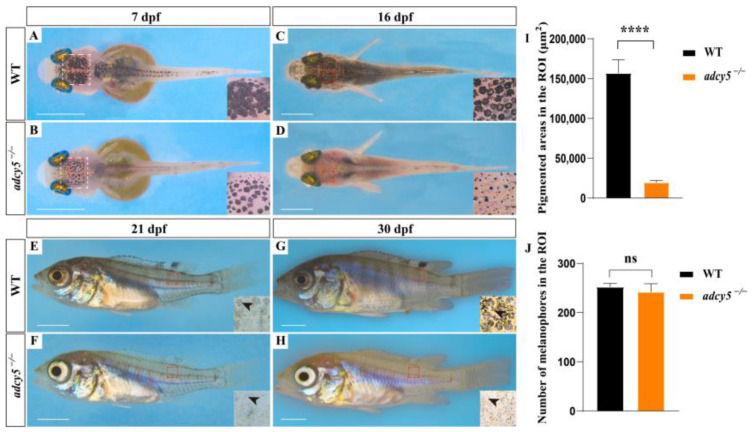
Phenotypes of WT and *adcy5* homozygous mutants at 7–30 dpf. (**A**,**B**) At 7 dpf. (**C**,**D**) At 16 dpf. (**E**,**F**) At 21 dpf. (**G**,**H**) At 30 dpf. Scale bar in (**A**–**H**), 1 mm. Red boxes indicate the magnified areas shown in the adjacent panels. (**I**,**J**) Statistical analysis of the coverage areas and the number of melanophores in WT and *adcy5*^−/−^ mutants at 7 dpf, with the head region of interest (ROI) indicated by the white box. Data are expressed as the mean ± SD (*n* = 3). (Student’s *t*-test, ****, *p* < 0.0001, ns, not significant, *p* > 0.05). dpf, days post-fertilization.

At 7 dpf, melanophore distribution along the head and dorsal axis was similar between WT and *adcy5*^−/−^ mutants. However, while melanophores in WT were stellate and dispersed, those in the mutants exhibited a compact, punctate morphology (Figure 3A,B). Quantitative analysis of region of interest (ROI) on the head demonstrated a significantly smaller pigmented area in *adcy5*^−/−^ than in WT fish, which was consistent with the aggregated melanophore morphology observed in *adcy5*^−/−^ fish (Figure 3I). However, no significant difference in melanophore number within the ROI was detected between WT and *adcy5*^−/−^ fish (Figure 3J). By 16 dpf, this morphological divergence intensified, with melanophores further dispersed across the head and dorsal regions in WT fish, while more contracted in *adcy5*^−/−^ mutants (Figure 3C,D). By 21 and 30 dpf, phenotypic differences became more pronounced. At 21 dpf, melanophores in WT formed distinct, band-like aggregates at the dorsal fin edges, while *adcy5*^−/−^ mutants displayed faded patches associated with sustained melanophore aggregation and reduced pigmentation (Figure 3E,F). At 30 dpf, *adcy5*^−/−^ mutants failed to develop the vertical black bars observed in WT fish, and exhibited a pale-yellow appearance (Figure 3G,H).

#### 3.5.2. Late Maintenance Phase (90 Dpf to Adulthood): Pigment Dispersion and Stable Body Coloration

**Figure 4:**
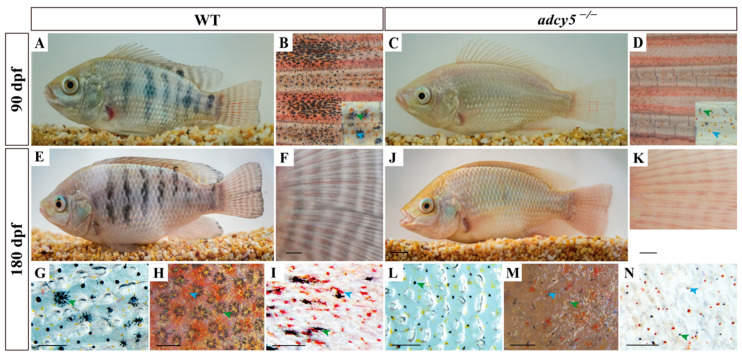
Phenotypes of WT and *adcy5* homozygous mutants at 90 and 180 dpf. (**A**,**C**) Overall phenotype of WT and *adcy5*^−/−^ mutants (90 dpf). Compared to WT, *adcy5*^−/−^ mutants lack black vertical stripes on both the trunk and fin rays. (**B**,**D**) Phenotype of the caudal fin in WT and *adcy5*^−/−^ mutants. Compared to WT, melanophores and erythrophores in the caudal fin of *adcy5*^−/−^ mutants present a punctate morphology. Scale bar in (**A**,**C**), 1 cm; scale bar in (**B**,**D**), 0.25 mm. dpf, days post-fertilization. (**E**,**F**,**J**,**K**) Phenotypes of whole fish and caudal fin in WT and *adcy5*^−/−^ mutants (180 dpf). (**G**–**N**) Microscopic imaging of localized regions of scales, skin, and the caudal fin in WT and *adcy5*^−/−^ mutants. Scale bars: 1 cm in (**E**,**J**); 0.5 cm in (**F**,**K**); 0.1 mm in (**G**–**I**,**L**–**N**). dpf, days post-fertilization.

At 90 dpf, WT fish exhibited well-defined vertical black bars on the trunk, dorsal fin, and caudal fin. Within the caudal fin bars, both melanophores and erythrophores displayed a dendritic morphology, indicative of normal pigment dispersion in these chromatophore types (Figure 4A,B). In contrast, *adcy5*^−/−^ mutants failed to develop the distinct black bars observed in WT fish. Moreover, the dendritic chromatophores observed in WT fish were completely replaced by punctate chromatophores in the mutants (Figure 4C,D), suggesting that pigment dispersion was severely impaired in both melanophores and erythrophores because of *adcy5*^−/−^ deficiency. Notably, despite these pronounced morphological alterations, no obvious reduction in chromatophore abundance was observed in *adcy5*^−/−^ mutants.

To further characterize the effects of *adcy5* deficiency on body coloration, we examined body color patterning in 6-month-old adult WT and *adcy5*^−/−^ fish. Consistent with the phenotype observed at 90 dpf, WT fish displayed well-defined vertical black bars on both the trunk and caudal fin (Figure 4E,F). In contrast, *adcy5*^−/−^ mutants failed to develop black bars in either region (Figure 4J,K). Owing to impaired melanosome dispersion, which resulted in the overall contraction of melanophores and a relative increase in the visibility of xanthophores, *adcy5*^−/−^ mutants exhibited a light-yellow body coloration.

High-magnification observations of scales, skin, and caudal fins further revealed marked differences in chromatophore morphology between WT and *adcy5*^−/−^ fish. In WT fish, both melanophores and erythrophores exhibited normal pigment granule dispersion and a characteristic dendritic morphology. In contrast, pigment granules in *adcy5*^−/−^ mutants were aggregated, causing both melanophores and erythrophores to contract into punctate morphology. Moreover, melanophores in *adcy5*^−/−^ mutants exhibited noticeably lighter pigmentation than those in WT fish. Despite these pronounced pigmentation defects, no significant differences in survival or growth were detected between *adcy5*^−/−^ mutants and WT fish.

### 3.6. Adcy5 Mutation Affects Melanin Synthesis

**Figure 5:**
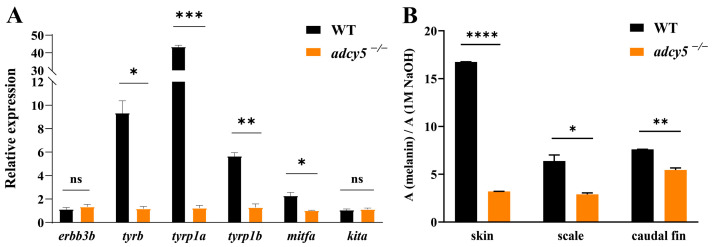
The effects of *adcy5* mutation on melanophore lineage differentiation and melanin synthesis. (**A**) qPCR detection of genes related to melanophore differentiation and melanin synthesis. (**B**) Quantification of melanin in the skin, scales, and caudal fins of WT and *adcy5*^−/−^ mutants at an absorbance of 490 nm. Data are expressed as the mean ± SD (*n* = 3). Significant differences in the data were tested by Student’s *t*-test. *, *p* < 0.05; **, *p* < 0.01; ***, *p* < 0.001; ****, *p* < 0.0001; ns, indicates no significant difference (*p* > 0.05).

The qPCR results showed that there were no significant differences in the expression level of *erbb3b* between *adcy5*^−/−^ and WT fish, indicating that *adcy5* mutation does not affect MSC fate determination. In contrast, the expression levels of *mitfa* and the melanogenesis-related genes *tyrb*, *tyrp1a*, and *tyrp1b* were significantly reduced in *adcy5*^−/−^ skin compared with WT skin (Figure 5A). Consistently, melanin quantification showed that melanin content in the skin, scales, and caudal fins was significantly lower in *adcy5*^−/−^ mutants than in WT fish (Figure 5B). These results indicated that loss of *adcy5* reduced melanin production, likely through downregulation of melanogenesis-related genes.

### 3.7. cAMP Treatment Partially Rescues the Melanosome Dispersion Defect in adcy5^−/−^ Mutants

**Figure 6:**
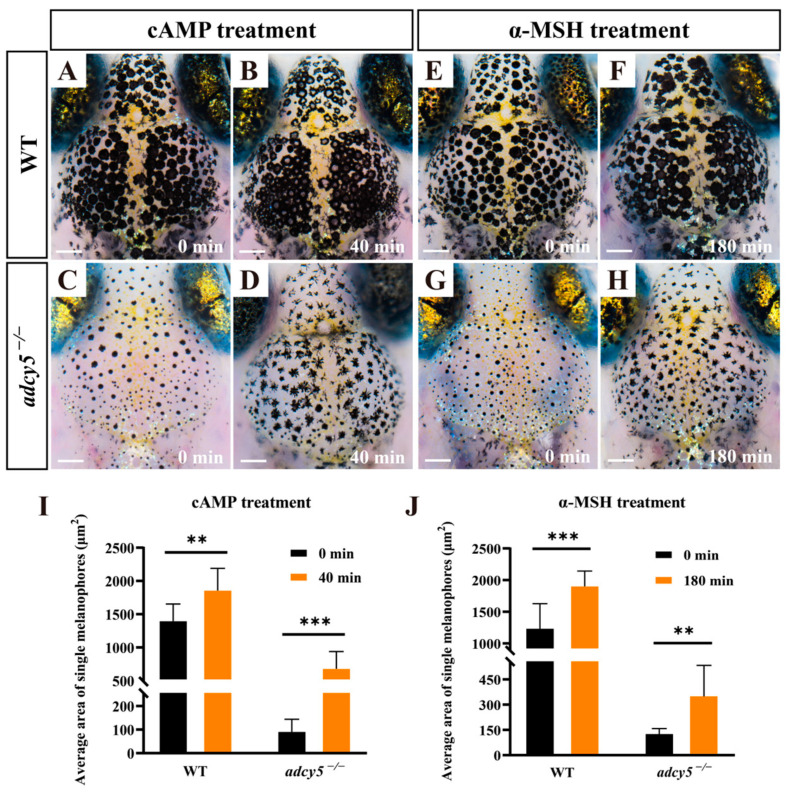
cAMP treatment, instead of α-MSH treatment, partially rescued melanosome dispersion in *adcy5*^−/−^ mutants. (**A**–**D**) After treatment with cAMP for 40 min, melanosomes in both WT and *adcy5*^−/−^ mutants showed significant dispersion. (**E**–**H**) After treatment with α-MSH for 180 min, melanosomes in melanophores of *adcy5*^−/−^ mutants exhibited mild dispersion, whereas in WT, melanosomes in almost all melanophores showed significant dispersion. (**I**,**J**) Statistical analysis of the average pigmented area of single melanophores in WT and *adcy5*^−/−^ mutants before and after treatment with cAMP and α-MSH, respectively. Data are expressed as the mean ± SD (*n* = 3). Scale bar in (**A**–**H**), 0.1 mm. Significant differences in the data were tested by Student’s *t*-test. **, *p* < 0.01, ***, *p* < 0.001.

Because ADCY proteins catalyze cAMP production, we next examined whether activation of cAMP-dependent signaling could alleviate the melanosome dispersion defect in *adcy5*^−/−^ mutants. After cAMP treatment, melanosomes were markedly dispersed in both WT and *adcy5*^−/−^ melanophores. By contrast, α-MSH treatment induced robust melanosome dispersion in WT melanophores but only mild dispersion in *adcy5*^−/−^ melanophores. Quantification of pigmented area of individual melanophores further confirmed that cAMP treatment significantly improved melanosome dispersion in *adcy5*^−/−^ mutants, whereas the response to α-MSH was strongly attenuated. Together, these results suggest that loss of *adcy5* impairs melanogenesis and attenuates α-MSH-induced melanosome dispersion, whereas direct cAMP treatment can partially rescue melanosome dispersion in *adcy5*^−/−^ melanophores (Figure 6).

Together, these findings support a model in which Adcy5 links α-MSH receptor activation to downstream cAMP-dependent signaling required for melanosome dispersion.

## 4. Discussion

### 4.1. Adcy5 Is a Conserved Regulator of Chromatophore Function

In guppies, mutation of *adcy5* abolishes melanosome dispersion in melanophores, resulting in complete pigment aggregation and loss of black stripe patterns [9]. This phenotype is consistent with our observations in Nile tilapia, where *adcy5* loss leads to similarly severe defects in pigment dispersion and bar formation. In zebrafish, *adcy5* single mutants display partial defects observed in guppies, with only a subset of melanophores exhibiting pigment aggregation, while others retain normal dispersion. As a consequence, residual stripe patterns are still detectable. In contrast, combined disruption of *adcy3a* and *adcy5* results in a more severe phenotype characterized by global pigment aggregation and complete loss of stripe patterning [10]. These observations suggest that Adcy5 represents a core component of the melanophore pigment dispersion machinery, whereas Adcy3a may provide partial compensatory activity in a species-dependent manner. In our study, loss of *adcy5* resulted in a striking pigmentation phenotype characterized by the absence of characteristic black vertical bars, reduced melanin coverage, lighter body coloration, and punctate melanophore morphology. These defects were observed from early larval stages and persisted into adulthood, indicating that Adcy5 is continuously required for melanophore pigmentation throughout development rather than functioning only during a specific developmental window. This suggests that Adcy5 has a conserved role in teleost melanophore pigmentation, but the degree of compensation by other ADCY members may vary among types.

### 4.2. Adcy5 Regulates Melanogenesis and Pigment Granule Transport Through cAMP-Dependent but Separable Outputs

Our results suggest that Adcy5 regulates chromatophore pigmentation through cAMP-dependent signaling that influences two partially separable downstream processes: melanogenesis and pigment granule transport.

At the transcriptional level, loss of *adcy5* was associated with reduced expression of *mitfa* and downstream melanogenic genes, including *tyrb*, *tyrp1a*, and *tyrp1b*, accompanied by decreased melanin content. This is consistent with a model in which cAMP–PKA signaling contributes to MITF-dependent transcriptional regulation of melanogenesis [23,24,25].

At the cellular level, *adcy5* mutants exhibited a pronounced aggregation of pigment granules in both melanophores and erythrophores, indicating impaired intracellular pigment transport. Importantly, chromatophore abundance remained unchanged, suggesting that Adcy5 primarily regulates functional pigment output rather than cell differentiation or survival. This phenotype is consistent with previous studies linking cAMP–PKA signaling to the regulation of microtubule-dependent motor activity, including kinesin- and dynein-mediated transport [26,27].

Together, these results support a model in which Adcy5 influences pigmentation through cAMP-dependent pathways that coordinate both gene expression programs and intracellular transport processes.

### 4.3. Adcy5 Likely Functions Upstream of Intracellular cAMP Production in the A-MSH Signaling Axis

Pharmacological assays provided functional support for the position of Adcy5 within the α-MSH signaling pathway. In wild-type melanophores, α-MSH stimulation induced robust pigment dispersion, consistent with activation of GPCR-dependent adenylate cyclase signaling.

In contrast, *adcy5* mutants showed a markedly reduced response to α-MSH, suggesting impaired signal transduction downstream of receptor activation. However, exogenous cAMP partially restored pigment dispersion in mutant melanophores, indicating that downstream signaling machinery remains functional.

These findings are consistent with a model in which Adcy5 acts upstream of intracellular cAMP production and is required for efficient coupling between receptor activation and cAMP generation. However, direct measurement of intracellular cAMP levels will be required to further validate this model.

### 4.4. Adcy5 Extends Its Regulatory Role to Erythrophore Pigment Granule Dispersion

A key finding of this study is that Adcy5 is also required for pigment granule dispersion in erythrophores, extending its functional role beyond melanophore regulation. In *adcy5* mutants, erythrophores exhibit a punctate morphology like that observed in melanophores, suggesting a shared requirement for Adcy5-dependent signaling in pigment granule transport across multiple chromatophore types. Although the molecular mechanisms of pigment transport in erythrophores remain less well characterized, previous studies in other teleost species indicate that pigment movement is dependent on microtubule-based transport systems [28]. Similar bidirectional transport mechanisms involving kinesin and dynein have been described in other intracellular granule systems [29,30], suggesting that conserved motor-based transport principles may operate in erythrophores as well.

Based on these observations, we propose that Adcy5-dependent cAMP signaling may regulate a conserved intracellular transport module across chromatophore types, although the precise downstream effectors in erythrophores remain to be defined.

### 4.5. A Unified Model for Adcy5-Mediated Regulation of Chromatophore Function

The tissue expression profile further supports the view that Adcy5 is not a pigmentation-specific factor, but rather a broadly expressed component of cAMP-dependent signaling. In adult Nile tilapia, *adcy5* showed the highest expression in the pituitary, with detectable expression in multiple peripheral tissues. This pattern suggests that Adcy5 may participate in both neuroendocrine and peripheral signal transduction. Nevertheless, the pigmentation phenotype observed in *adcy5* mutants is more directly attributable to impaired cAMP-dependent pigment granule dispersion in chromatophores, as supported by the punctate morphology of melanophores and erythrophores and the partial rescue of melanosome dispersion by exogenous cAMP.

Together, our findings support a model in which Adcy5 acts as a central component of the cAMP signaling network controlling chromatophore function. In this model, Adcy5 couples extracellular GPCR signals, such as α-MSH, to intracellular cAMP production, thereby coordinating both melanogenesis and pigment granule transport.

This dual regulatory architecture enables synchronized control of pigment synthesis and spatial distribution, ensuring robust pigment pattern formation. The extension of this mechanism to erythrophores further suggests that Adcy5 functions as a shared regulatory node across multiple chromatophore types in teleost fish.

## 5. Conclusions

This study identifies Adcy5 as a conserved regulator of chromatophore pigmentation in Nile tilapia. Loss of *adcy5* disrupts body coloration, leading to loss of vertical bars and aggregation of pigment granules in chromatophores. Mechanistically, Adcy5 regulates melanogenesis and pigment granule transport through cAMP-dependent signaling, as supported by reduced expression of key melanogenic genes and partial rescue by cAMP treatment. Importantly, Adcy5 is also required for pigment granule dispersion in erythrophores, suggesting a shared cAMP-dependent mechanism across multiple chromatophore types. In summary, this study establishes Adcy5 as an essential regulator of pigment granule dispersion in both melanophores and erythrophores. The Nile tilapia model, with its complete chromatophore repertoire, provides unique opportunities to dissect cell-type-specific regulatory mechanisms. Future studies should investigate the specific downstream targets of PKA signaling in different chromatophore types, and explore whether other ADCY isoforms regulate xanthophore and iridophore pigmentation.

## Data Availability

The original contributions presented in this study are included in the article and Supplementary Materials. Further inquiries can be directed to the corresponding authors.

## Supplementary Materials

The following supporting information can be downloaded at: https://www.mdpi.com/article/10.3390/cells15151347/s1, Figure S1: Synteny analysis of *adcy5* gene in vertebrates; Figure S2: Amino acid sequence similarity analysis of Adcy5 in vertebrates; Figure S3: Phylogenetic tree of Adcy5 in animals; Table S1: The accession numbers of the sequences used in this study; Table S2: Primer sequences used for qPCR in the present study; Table S3: The primer sequences used for the homologous line establishment of *adcy5*^−/−^.
