# Supplementary material for: Adenylate Cyclase 5 (Adcy5) Deficiency Impairs Pigment Granule Dispersion in Melanophores and Erythrophores in Nile Tilapia
Source: Cells. 2026 Jul 27;15(15):1347. doi: 10.3390/cells15151347 (PMC13465509; doi:10.3390/cells15151347)
Supplement: Supplementary file 1 [file cells-15-01347-s001.zip › Supplementary File.pdf]

Supplemental data

Tables

Table S1 The accession numbers of the sequences used in this study

Gene	Accession Number	Gene	Accession Number
Ol-adcy5	XP_020568498.1	*Lc-adcy5*	XP_064420007.1
Ta-adcy5	XP_002111246.1	*Lo-adcy5*	XP_015213942.1
Hv-adcy5	XP_047136993.1	*On-adcy5*	XP_013132811.1
Dr-adcy5	NP_001165056.1	*Gg-adcy5*	NP_989962.2
Cg-adcy5	XP_011453239.3	*Bb-adcy5*	KAI8515270.1
Dm-adcy5	NP_730173.3	*Pm-adcy5*	XP_032821389.1
Ci-adcy5	XP_026693427.1	*Tr-adcy5*	XP_011611519.1
Cm-adcy5	XP_042192120.1	*Zv-adcy5*	XP_034984880.1
Hs-adcy6	NP_056085.1	*Hs-adcy5*	NP_001365188.1

On, Oreochromis niloticus; Tr, Takifugu rubripes; Dr, Danio rerio; Lo, Lepisosteus oculatus; Gg, Gallus gallus; Hs, Homo sapiens; Lc, Latimeria chalumnae; Pm, Petromyzon marinus; Bb, Branchiostoma belcheri; Cg, Crassostrea gigas; Ta, Trichoplax adhaerens; Hv, Hydra vulgaris; Dm, Drosophila melanogaster; Ci, Ciona intestinalis; Ol, Oryzias latipes; Zv, Zootoca vivipara; Cm, Callorhinchus milii

Table S2 Primer sequences used for qPCR in the present study

Primer	Sequence (5'-3')
erbb3b-qPCR-F	CTCAACTACCCCAAGGACGG
erbb3b-qPCR-R	CTGCCAGAAGATCCAGGGAC
mitfa-q-F	AAGCCTCGGTGGACTACATT
mitfa-q-R	CAACTCCTGTATACGGAGCATA
tyrb-q-F	TCATCAGCCCTGCCTCTGTCTT
tyrb-q-R	AAGTCGTTGCACGCGATTGGT
tyrp1a-q-F	TCATCAGCCCTGCCTCTGTCTT
tyrp1a-q-R	GAAGACTCAGTGCTGTTGCAG
tyrp1b-q-F	GCGATGCAGACATGGGCTA
tyrp1b-q-R	AACAACTCCTGGTACCGTCT
kita-q-F	ATTCAATTAAGAAGGCTCTGCC
kita-q-R	TTCACATAGACGTAGATGGAGG
β-actin-q-F	CATCCCGTCCTGCTCACA
β-actin-q-R	AGGCGTACAGGGACAGCA

Table S3 The primer sequences used for the homologous line establishment of *adcy5*$^{-/-}$

Primer	Sequence （5'-3'）
adcy5-gRNA-F	TAATACGACTCACTATAGGACCGAGCACAGATCA GCTGTTTTAGAGCTAGAAATAGC
gRNA-R	AGCACCGACTCGGTGCCAC
adcy5-page-F	CATTCTGTCACTGCATCCACTT
adcy5-page-R	TGAGTATGGGCATCCGTTGG
adcy5-test-F	CGCCTCATCCTTGGGACTAA
adcy5-test-R	TTGTTGTCTCGGCTCTCCTC

Figures

Figure S1

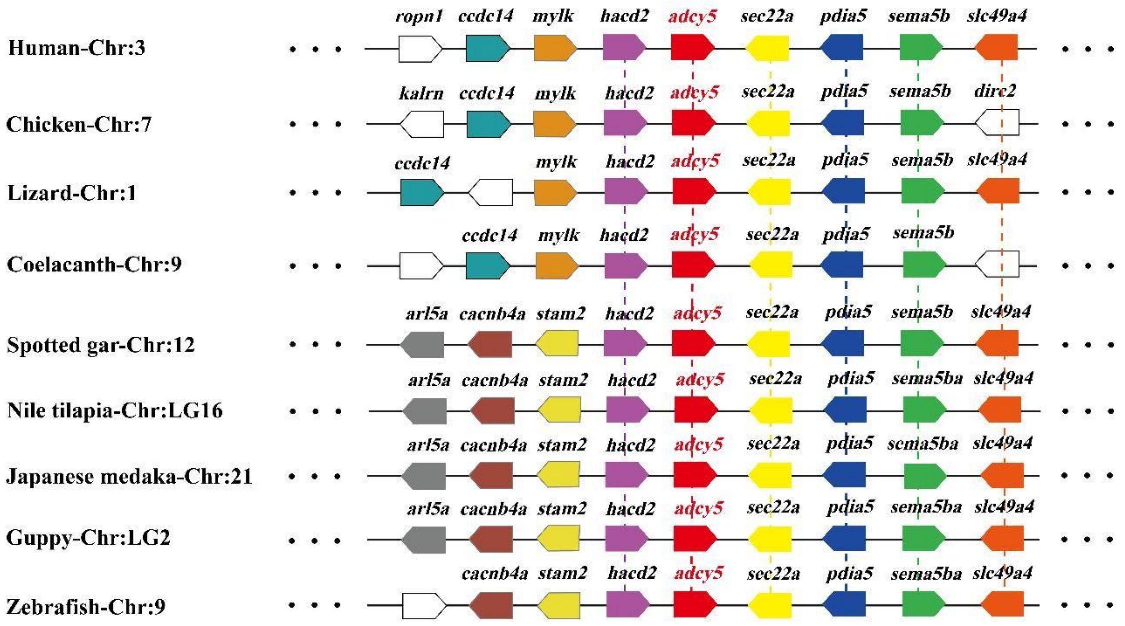

Figure S1 Synteny analysis of *adcy5* gene in vertebrates

Different colored blocks represent different genes, with the direction of the arrows indicating the gene orientation on the chromosome. White blocks represent unannotated genes. "Chr" stands for chromosome, and "LG" stands for linkage group.

Figure S2

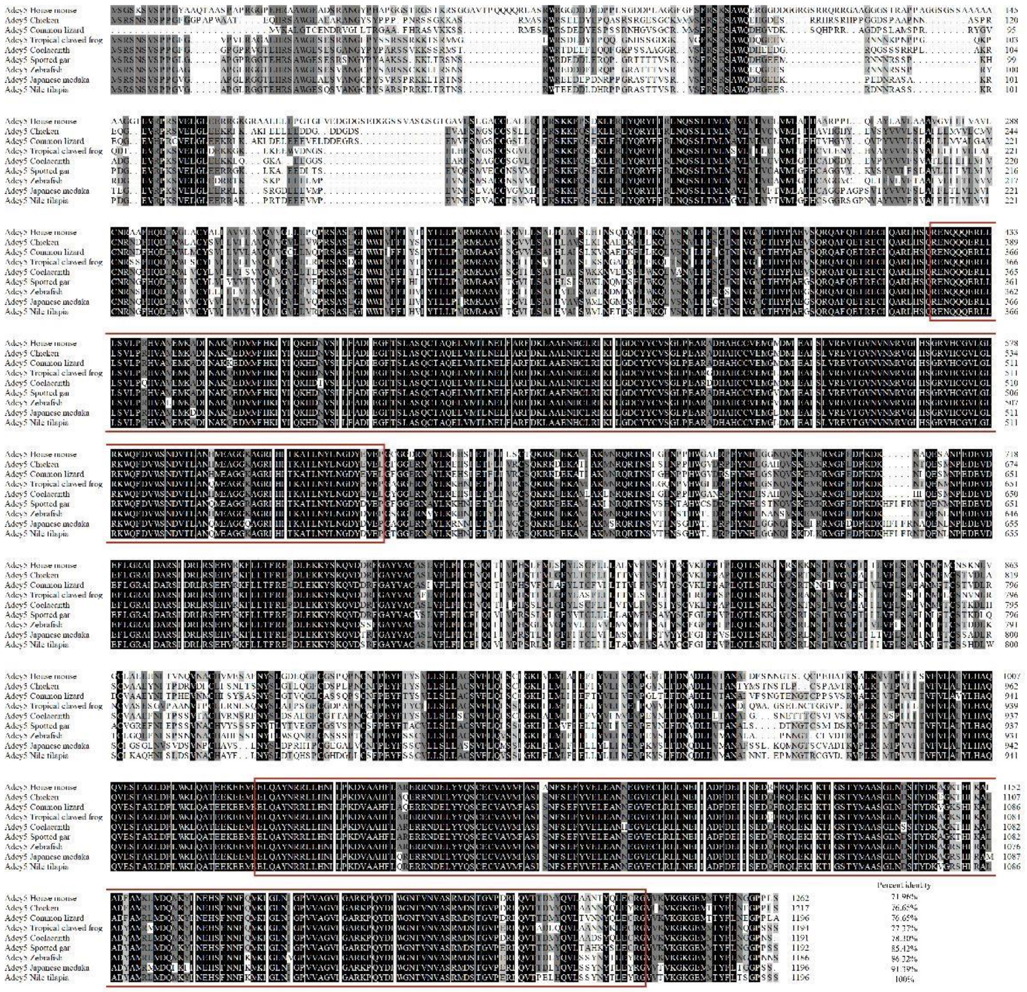

Figure S2 Amino acid sequence similarity analysis of Adcy5 in vertebrates

The amino acid sequence alignment map was generated using DNAMAN software. Black regions indicate fully conserved amino acids, dark gray regions represent amino acids with a homology level of 75%≤x<100%, and light gray regions indicate a homology level of 50%≤x<75%. The red box represents the catalytic domain. The percentages indicate the degree of amino acid sequence identity between Adcy5 of Nile tilapia and that of other species.

Figure S3

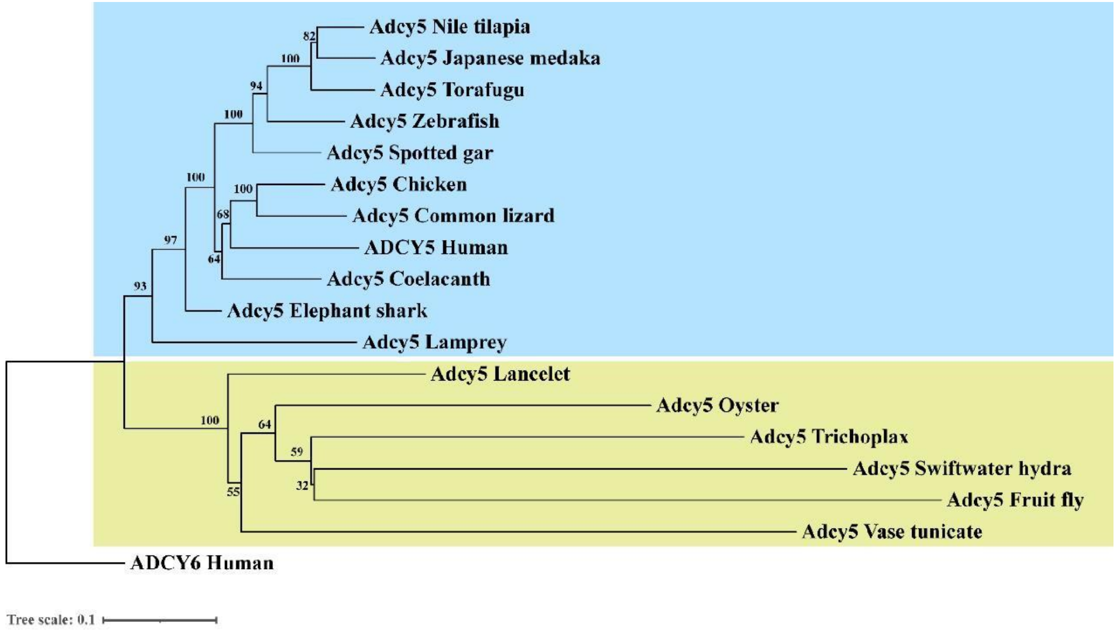

Figure S3 Phylogenetic tree of Adcy5 in animals

The ML method was used to construct the tree by PhyloSuite software. The multiple sequence alignment of amino acids is based on the MAFFT algorithm.
